# An eggshell-localised annexin plays a key role in the coordination of the life cycle of a plant-parasitic nematode with its host

**DOI:** 10.1371/journal.ppat.1011147

**Published:** 2023-02-13

**Authors:** James A. Price, Mohammad Farhan Ali, Louise L. Major, Terry K. Smith, John T. Jones

**Affiliations:** 1 Cell & Molecular Sciences Department, The James Hutton Institute, Invergowrie, Dundee, United Kingdom; 2 School of Biology, Biomedical Sciences Research Complex, University of St. Andrews, North Haugh, St. Andrews, United Kingdom; University of California, Davis Genome Center, UNITED STATES

## Abstract

Host-specific plant pathogens must coordinate their life cycles with the availability of a host plant. Although this is frequently achieved through a response to specific chemical cues derived from the host plant, little is known about the molecular basis of the response to such cues and how these are used to trigger activation of the life cycle. In host-specific plant-parasitic cyst nematodes, unhatched juvenile nematodes lie dormant in the eggshell until chemical cues from a suitable host plant are detected and the hatching process is initiated. The molecular mechanisms by which hatch is linked to the presence of these chemical cues is unknown. We have identified a novel annexin-like protein that is localised to the eggshell of the potato cyst nematode *Globodera rostochiensis*. This annexin is unique in having a short peptide insertion that structural modelling predicts is present in one of the calcium-binding sites of this protein. Host-induced gene silencing of the annexin impacts the ability of the nematode to regulate and control permeability of the eggshell. We show that in the presence of the chemicals that induce hatching annexin lipid binding capabilities change, providing the first molecular link between a nematode eggshell protein and host-derived cues. This work demonstrates how a protein from a large family has been recruited to play a critical role in the perception of the presence of a host and provides a new potential route for control of cyst nematodes that impact global food production

## Author summary

Potato cyst nematodes (PCN) are pathogens of economically important solanaceous plants. Like other host-specific plant-pathogens, PCN resume their life cycle specifically in response to chemical cues from suitable host plants. Here, for the first time, we describe, an eggshell localised annexin that plays a key role in the response to the presence of secreted host chemicals. Reducing the level of this protein impacts the ability of the nematode to control permeability of the eggshell, while the lipid binding properties of the protein change specifically in the presence of host derived chemical cues, ultimately affecting hatching. This work represents the first molecular determination of how a plant-parasitic nematode responds to a host chemical. Additionally, this is the first occurrence of identification and localisation of any parasitic nematode eggshell protein. The techniques used in this research can be widely applied to other species of nematodes allowing discovery of further eggshell-associated proteins and development of a new generation of nematicides or anthelminthics.

## Introduction

Many plant pathogens have a restricted host range and infect a relatively small number of taxonomically related host plants [1]. For these pathogens the ability to coordinate their life cycle with availability of a host plant is a critically important aspect of their biology. Many plant pathogens achieve this coordination with their hosts through a response to specific host-derived chemical cues. For example, zoospores of the oomycete soybean pathogen *Phytophthora sojae* are sensitive to isoflavones derived from the roots of soybeans whereas other species that do not infect these plants do not respond to these chemicals [2,3]. However, for most pathosystems, little is known about the molecular mechanisms by which these chemical cues are perceived and how the responses are activated.

Cyst nematodes are extremely damaging plant pathogens that infect a wide range of crop plants [4]. Many cyst nematode species have restricted host ranges and need to coordinate their life cycles with those of their host. This includes the potato cyst nematode (PCN), *Globodera rostochiensis*, which infects a restricted range of Solanaceous plants. These nematodes cause an estimated global potato crop loss of 9% and have recently been discovered in East Africa where they represent a significant barrier to achieving food security in the region [5,6,7].

Like other cyst nematodes, PCN moult to a dormant stage (the second stage juvenile–J2) which remains unhatched in the egg in a state of metabolic arrest until the presence of a host is detected [8]. The unhatched J2 is extremely well adapted for long-term survival in adverse environments. The eggshell, which is composed of numerous lipid layers as well as a thick chitinous layer [9], is selectively permeable and provides protection from biotic and abiotic stresses. Within the eggshell the unhatched J2 is partially dehydrated and surrounded by a vitelline fluid that contains a high concentration of trehalose, protecting the dormant nematode from extremes of temperature. The hatching and reactivation processes are stimulated by exposure to mixture of chemical cues contained within the root diffusates of host plants. Analysis of fractionated root diffusate has shown that hatching can be induced by several different compounds [10], and individual compounds that induce hatching, including α-solanine and solanoeclepin A, have been identified [11,12].

The physiological changes that occur during hatching of PCN are well documented [13]. Exposure to host root diffusates induces a change in the permeability of the eggshell, allowing trehalose to leave the vitelline fluid and water to diffuse in. This allows rehydration of the nematode and consequently the reactivation of nematode metabolism. Calcium (Ca^2+^) plays a key role in this process, with the change in permeability of the PCN eggshell in response to root exudates thought to involve a change in Ca^2+^ binding [13]. Hatching of broad host-range species of cyst nematodes, such as *Heterodera schachtii*, can be induced by exposure to divalent cations [14] but this is not the case for PCN [15].

Although hatching of plant-parasitic nematodes has been well described at a physiological level, the molecular mechanisms that underpin the hatching process remain relatively underexplored. Nothing is known about how root exudates are perceived by dormant PCN. Understanding the molecular basis of how pathogens perceive the presence of a host offers an opportunity for development of novel control strategies, by inducing hatch in the absence of a host. Here we describe an annexin-like protein localised to the eggshells of PCN that plays a role in the perception of the presence of a host. We show that reducing expression of the gene encoding this protein impacts the permeability of the eggshell and that the presence of host root diffusates results in differential lipid binding in a host specific manner. This work provides the first insights into the molecular mechanism by which a plant pathogen links its life cycle to host-derived cues.

## Results

### Identification of an eggshell annexin

Pure eggshell samples were obtained using a modified eggshell purification method (Fig A in S1 Data) allowing analysis of eggshell proteins with minimal contamination from unhatched juveniles. Proteins identified by mass spectrometry analysis of purified eggshells were compared across multiple experimental replicates (Fig B in S1 Data). No eggshell proteins have previously been identified from any parasitic nematodes. Two proteins (*G*. *rostochiensis* gene codes GROS_g06666 and GROS_g01373), sharing similarity to chondroitin proteoglycans (CPGs), were identified in these mass spectrometry data. CPGs have previously been identified as components of the *C*. *elegans* eggshell [16]. The presence of these proteins in the *G*. *rostochiensis* eggshell mass spectrometry dataset provided reassurance that the protocol being used allowed identification of genuine eggshell proteins.

**Fig 1:**
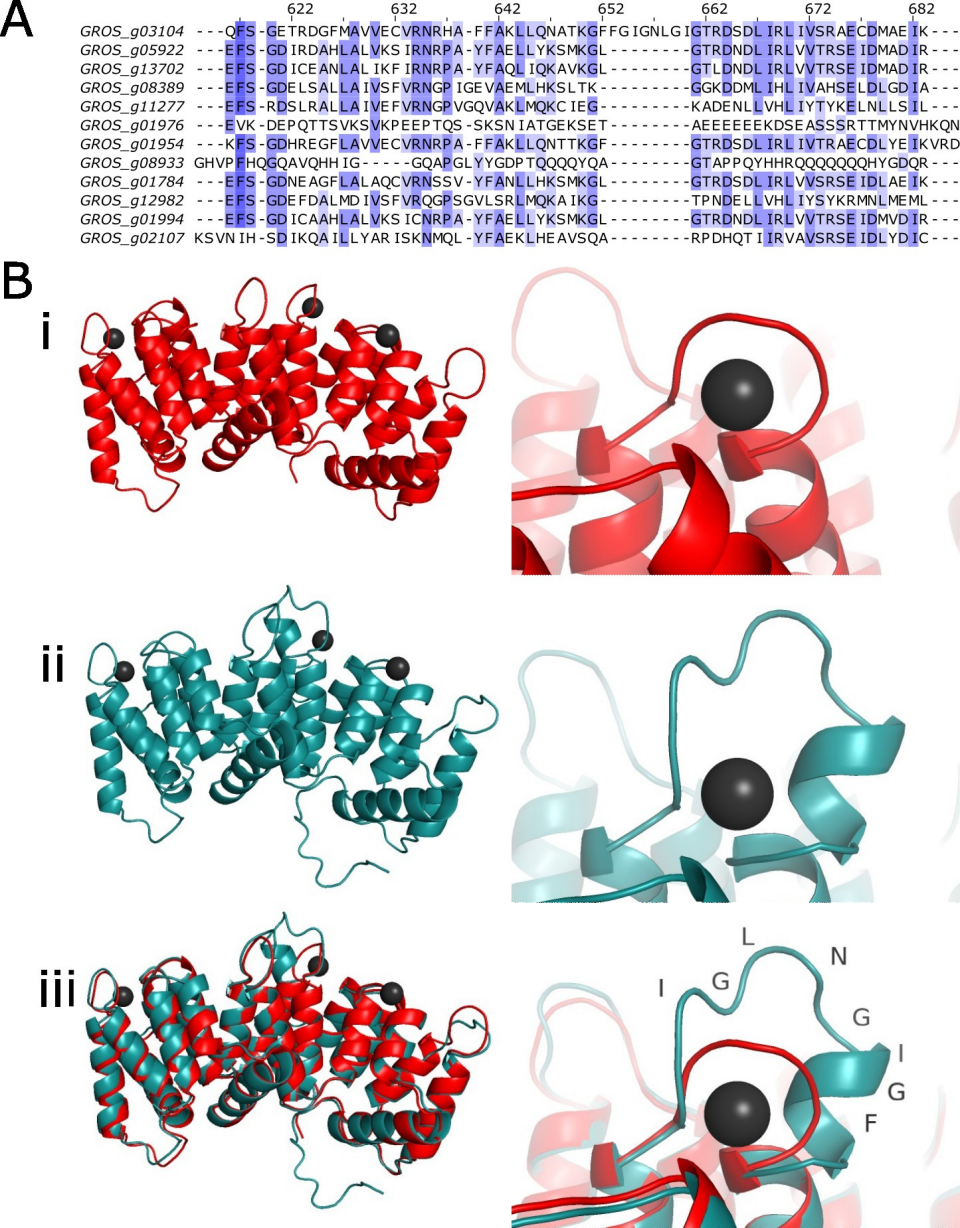
Annexin sequence and structure. (A) Alignment of *G*. *rostochiensis* annexin sequences. The annexin identified in mass spectrometry data (GROS_g03104) is distinguished by the unique peptide N-^267^FFGIGNLGI^275^-C. Blue colouring indicates conserved amino acids between proteins. (B) Structural prediction for GROS_g03104 from phyre2. (i), Crystal structure of bovine annexin VI PDB ID: 1AVC with black spheres representing calcium ions bound to 1AVC annexin structure. (ii), predicted structure for GROS_g03104 with focus on *Globodera spp*. unique insertion and overlay of calcium ions associated with bovine annexin 1AVC. (iii), overlay of PDB ID: 1AVC and the GROS_g03104 prediction.

Peptides from an annexin-like protein (GROS_g03104) were identified in all protein extraction replicates. Annexins are calcium-dependent lipid-binding proteins and, given the apparent dependency on a change in Ca^2+^ during the PCN hatching cascade and the importance of lipids in the nematode eggshell permeability barrier, GROS_g03104 was of obvious interest. Annexins are highly conserved proteins that are present as multigene families in all eukaryotic organisms. In keeping with this, BLASTP searches of the *G*. *rostochiensis* genome sequence identified 17 annexin-like proteins like GROS_g03104. However, the GROS_g03104 sequence contains a unique insertion (N-^267^FFGIGNLGI^275^-C) which is not present in any other *G*. *rostochiensis* annexins (Fig 1A). Furthermore, this unique insertion is not present in any other annexins identified from other organisms, except for one similar sequence in another closely related PCN species, *Globodera pallida* (Fig C in S1 Data). This peptide insertion was directly identified in the mass spectrometry analysis of *G*. *rostochiensis* eggshells, confirming that GROS_g03104 is the annexin identified in eggshell protein extractions. Cloning of the GROS_g03104 gene from *G*. *rostochiensis* cDNA confirmed that the transcript and protein sequence are correctly predicted. A structural prediction for GROS_g03104 was produced using the online resource Phyre2 [17]. The amino acid sequence for GROS_g03104 was modelled against the solved structure for the dimeric bovine annexin PDB ID: 1AVC [18] (Fig 1B). Monomeric annexin units for both GROS_g03104 and PDB ID: 1AVC share a large amount of structural similarity. The unique amino acid insertion is predicted to overlay one of the predicted calcium binding site distending the usual structure in this area. Models produced by AlphaFold 2 [19] produce near identical results, although the position of the unique motif is angled differently.

### Spatial and temporal expression patterns of the eggshell annexin

**Fig 2:**
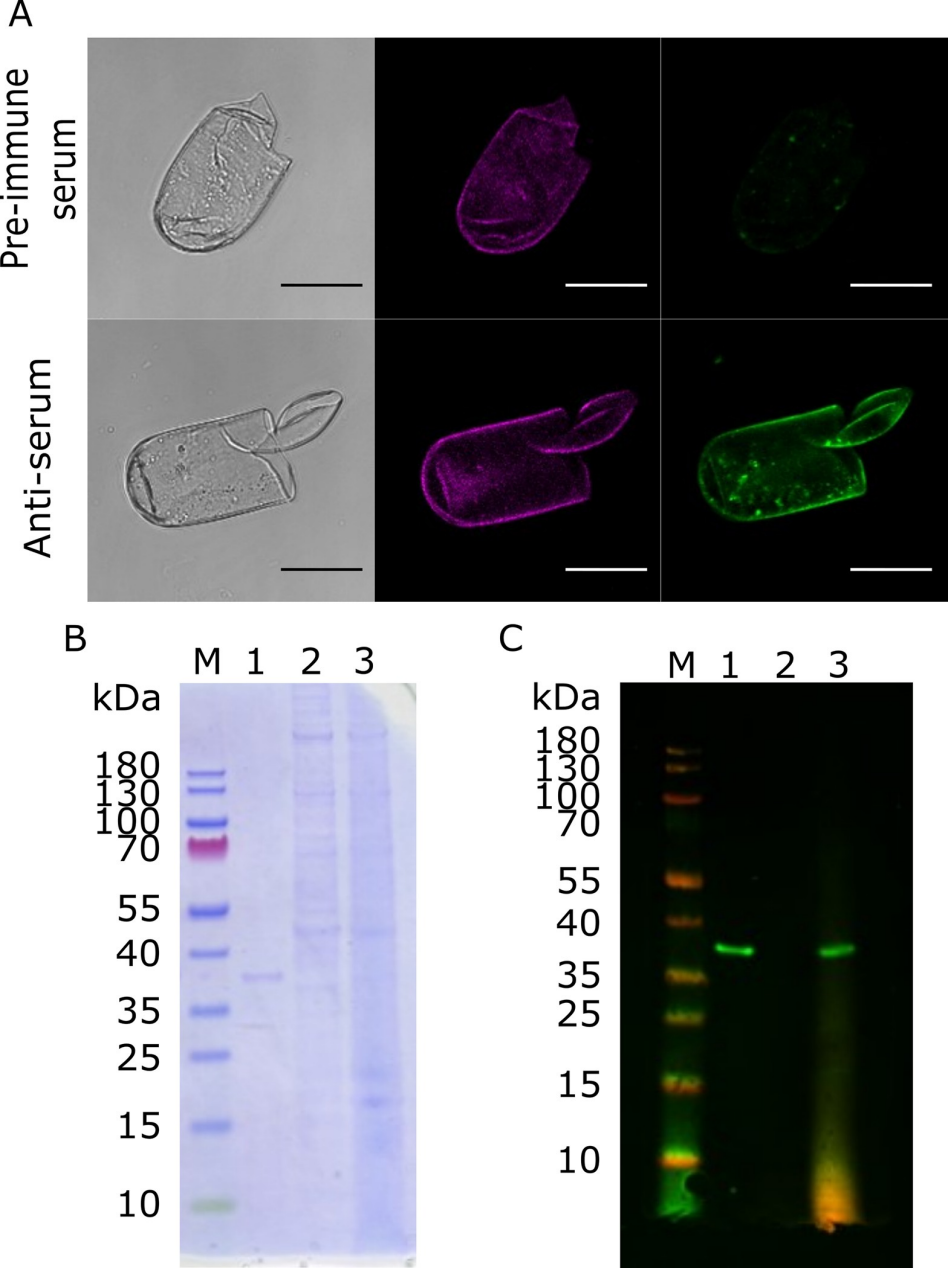
Annexin Localisation. A) Binding of antiserum raised against immunogenic, unique, peptides from GROS_g03104 on *G*. *rostochiensis* eggshell. Weak fluorescence can be seen in eggshells when imaging pre-immune serum samples due to native eggshell autofluorescence. Images left to right; DIC, laser line 516 nm, laser line 488 nm. Scale bar represents 50 μm. B) SDS-PAGE stained with coomassie. Samples are M–Marker (PageRuler Prestained Protein Ladder); 1 – 100ng recombinant annexin; 2—Juvenile (J2) *G*. *rostochiensis* lysate from (~20,000 J2s); 3 –*G*. *rostochiensis* cyst lysate (containing eggshells and juveniles, 30 cysts). C) Western blot for (B) with same sample layout. Primary antibody 1:5000 A1, secondary antibody 1:20000 Invitrogen Goat anti-Rabbit IgG Alexa Fluor Plus 800. GROS_g03104 antiserum binds to annexin only when eggshells are present, confirming specificity.

Antibodies were produced against unique immunogenic regions of the *G*. *rostochiensis* annexin and used for immunolocalisation of the annexin-like protein in eggshells. The antibody showed specific binding to recombinant GROS_g03104 produced in *E*. *coli* (Fig 2B). Clear binding of anti-annexin antibodies to *G*. *rostochiensis* eggshells was observed, with no binding seen for samples exposed to pre-immune serum (Fig 2A). This, in addition to western blotting of eggshell protein extractions (Fig 2C), confirmed that the annexin is present in the eggshells of *G*. *rostochiensis*. No binding of the antiserum to proteins extracted from J2 was observed (Fig 2C).

**Fig 3:**
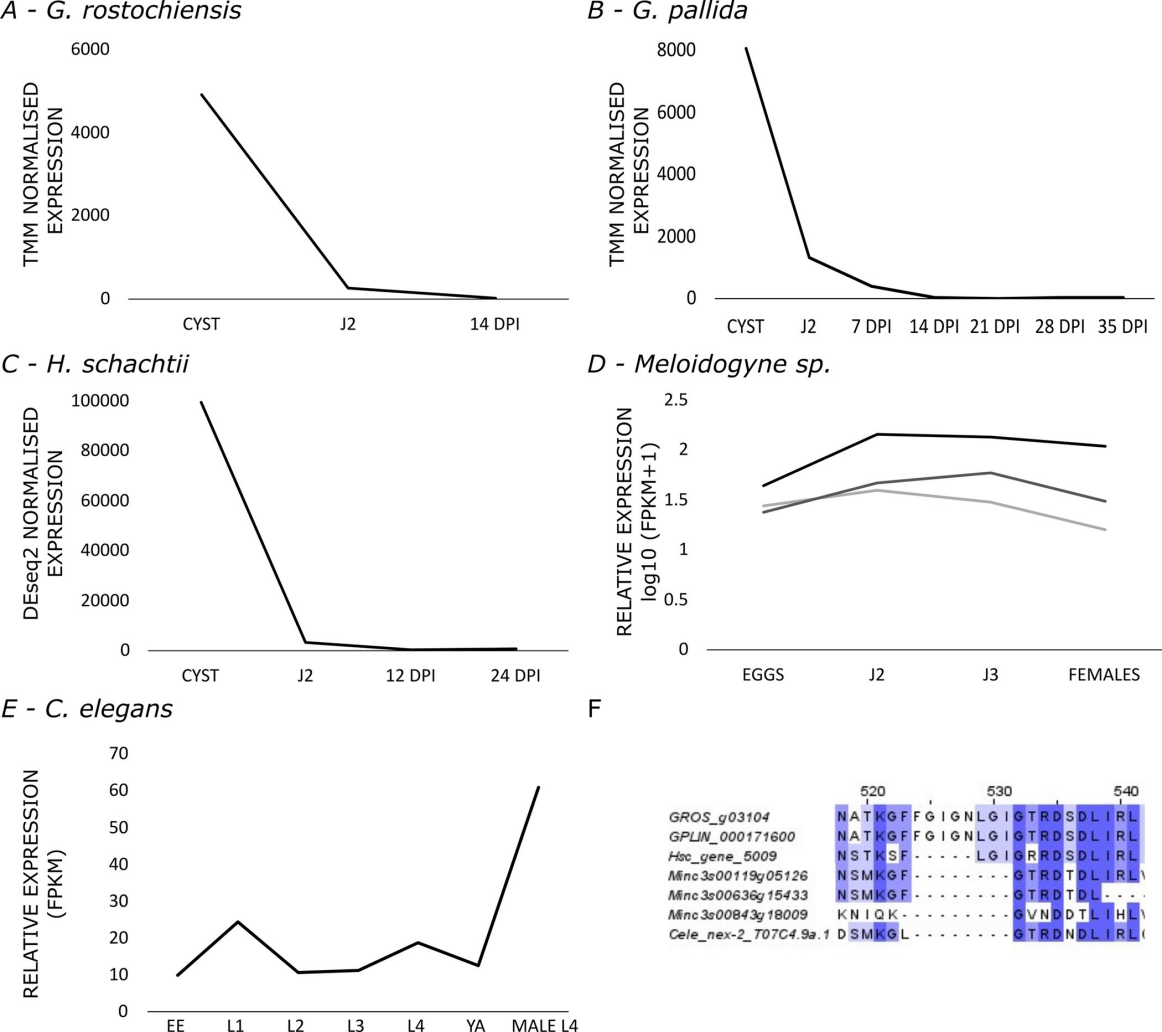
Eggshell annexin and orthologue expression. Life stage specific annexin and orthologue data showing high expression in the eggshell forming, cyst stages. Little to no expression of this protein is seen throughout the parasitic life stages for; (A) GROS_g03104 in *G*. *rostochiensis*. (B) GPLIN_000171600 in G. pallida. (C) Hsc_gene_5009 in H. schachtii, (D) Expression data for the three most similar M. incognita annexin sequences showing no upregulation at eggshell forming life stages. (E) *C*. *elegans* nex-2/T07C4.9a.1 (F) Sequence alignment showing region of interest containing unique Globodera insertion. Abbreviations: DPI: days post infection, J2/3: second/third stage juvenile, EE: early embryo, L1/2/3/4: larval stages 1–4, YA: young adult.

Analysis of RNAseq data for *G*. *rostochiensis* [20] and *G*. *pallida* [21] showed that GROS_g03104 and the top BLAST match in *G*. *pallida* (GPLIN_000171600) are both abundantly expressed at egg producing life stages with little or no expression in the hatched J2 or during parasitic life stages (Fig 3A and 3B). This expression profile was not seen for any other *G*. *rostochiensis* or *G*. *pallida* annexin sequences. This stage-specific expression pattern was shared by the orthologous protein in *Heterodera schachtii* which shares part of the *Globodera spp*.-specific annexin motif (Fig 3C and 3F) and may therefore have a similar functional role [22]. The three most similar sequences in the root knot nematode *Meloidogyne incogntia* (Fig 3D) [23] and orthologous protein in the free-living nematode *C*. *elegans* (Fig 3E) [24] do not share a similar expression profile and lack any insertion at the region of interest. These differences in expression profile may therefore reflect an adaption for a specific function in the eggshell.

### RNA mediated knockdown of the eggshell annexin disrupts the ability of the nematode to control eggshell permeability

Creation of stable PCN transgenic lines is not currently possible. Therefore, host mediated RNAi was used to knock down expression of the GROS_g03104 mRNA. For this, transgenic lines of potatoes expressing short hairpin RNA (shRNA) targeting GROS_g03104 mRNA were produced. These shRNAs are taken up by the parasitic stage nematode when feeding; this strategy has been used to analyse function of several PPN genes (*e*.*g*., [25]). Lines transformed with an empty vector (EV) were used as a negative control. After 7 weeks of growth, 5 mature females were extracted from 3 random cuttings of the plant lines. qPCR on mature female cDNA was used to confirm eggshell annexin knock down compared to the expression of the unaffected housekeeping gene GAPDH (Fig F in S1 Data).

**Fig 4:**
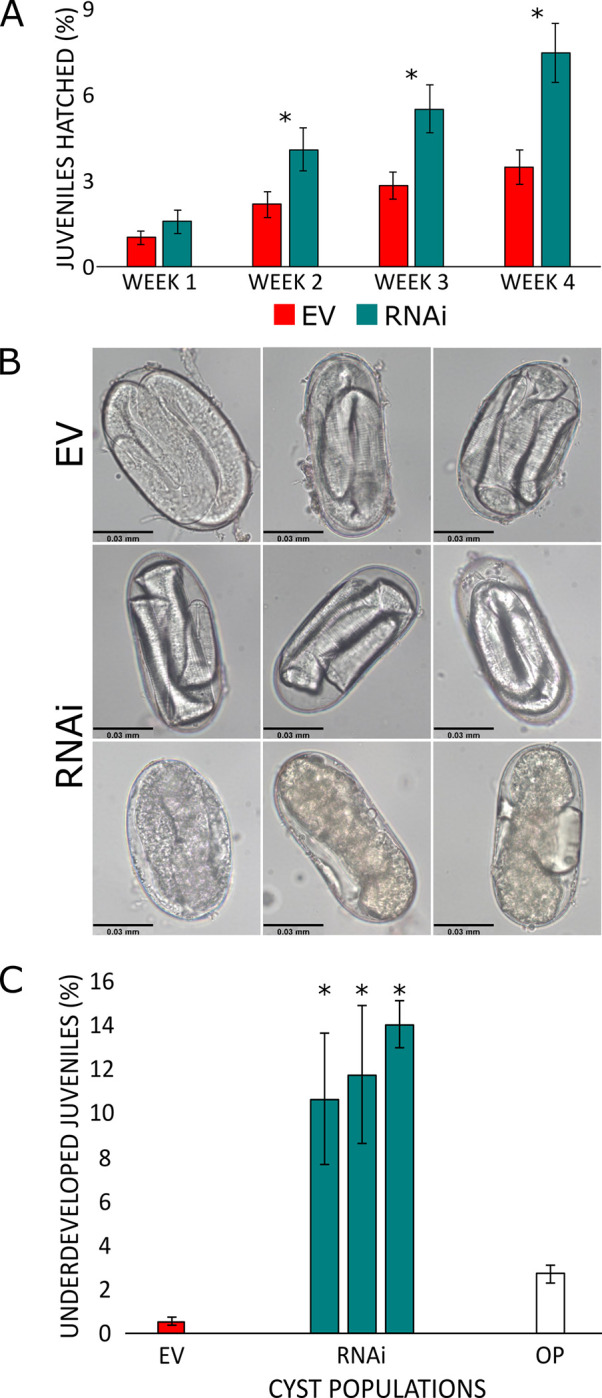
Annexin RNAi and effects on nematode hatching. (A) Average percentage of juveniles hatched per week. A significant increase in hatching in annexin RNAi lines (p<0.05) was visible after 2 weeks, marked with ‘*’. (B) *G*. *rostochiensis* developmental changes after RNAi. Top–negative control lines using an empty vector (EV), juveniles are swollen and fill the entire eggshell. Middle- juveniles inside eggs following RNAi showing larger space in the perivitelline area between juvenile and eggshell. Bottom–eggs collected from cysts exposed to HIGS, no distinguishable juvenile morphological features are present suggesting interrupted development. (C) Percentage of underdeveloped juveniles found in negative control or RNAi cyst populations. There is a significant increase in underdeveloped juveniles in cysts populations that have reduced annexin availability as marked with ‘*’. Cyst populations were also compared to a sample taken from the original population of cysts used to inoculate transgenic plants (OP).

Hatching assays were used to identify how eggshell annexin knock down affected nematode hatching in response to host root diffusates. Nematodes harvested at this stage would normally be expected to show very low hatch in response to root diffusates as they have not been exposed to a period of cold to break diapause. Hatching of *G*. *rostochiensis* juveniles was monitored over a 4-week period after a single exposure to root diffusates. Averages were calculated across lines that either did or did not show annexin knock down (Fig 4A). Unaveraged hatching data per cyst population showed that cysts from transgenic lines with the largest decrease in fold change for annexin expression showed the highest levels of hatching in response to host root diffusates (Figs F and H in S1 Data). Nematodes that hatched in these experiments were motile and appeared to have normal J2 morphology.

Due to the hierarchical assembly of nematode eggshells, disruption of eggshell formation can result in phenotypes showing developmental failures [26]. Therefore, unusual phenotypes in nematode eggs developing in females exposed to the shRNAs were monitored. No impact was observed on the development of female nematodes on the transgenic plants. There were no overall differences in diameter of cysts that had been raised on shRNA expressing lines compared to control lines (Fig G in S1 Data). Juveniles developing within eggshells from cysts collected from negative control plants showed no unusual phenotype. The juveniles developed normally and had grown to fill the available space within the eggshell (Fig 4B). However, a noticeable proportion of juveniles in eggshells from host lines expressing the shRNA did not fully grow to fill the eggshell, looking smaller and dehydrated (Fig 4B). Additionally, a significant (*p* < 0.001) percentage of the juveniles appeared to have delayed development completely, with little distinguishable body morphology (Fig 4B and 4C). These data show that reducing expression of the annexin produces phenotypical effects that suggest an impact on the ability of the nematode to control permeability of the eggshell.

### A calcium mediated change in conformation of the annexin is affected by the presence of host root exudates

**Fig 5:**
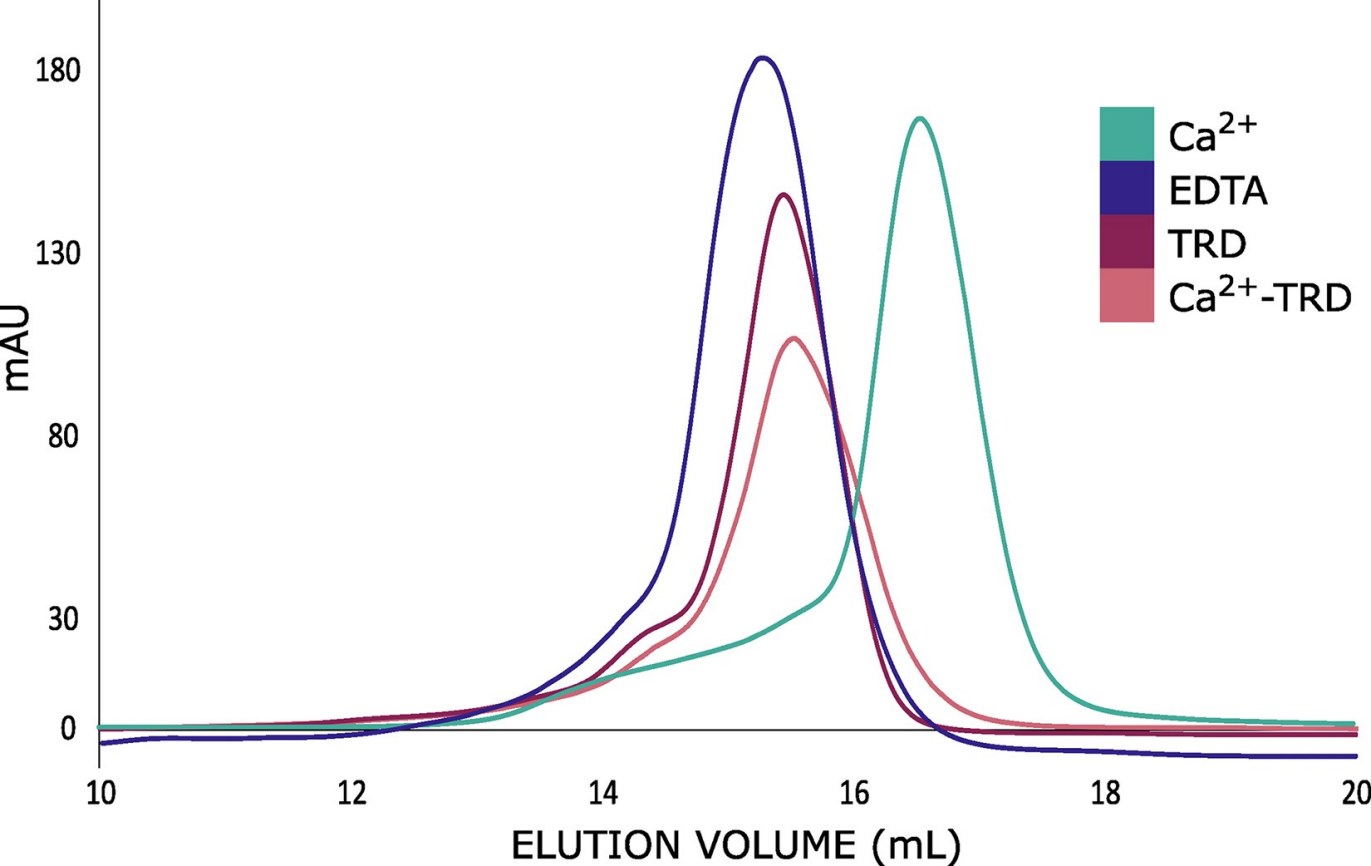
Annexin-host interaction. Shift in elution from a Superose 6 Increase 10/30 analytical column for recombinant annexin in response to 1 mM CaCl_2_ or 1 mM EDTA. In the presence of host root diffusates (TRD), annexin no longer shifts to the calcium bound state.

The online resource 3DLigandSite [27] predicts multiple calcium binding sites in the GROS_g03104 protein, one of which is potentially disrupted by the *Globodera spp*. specific motif. Size exclusion chromatography (SEC) using recombinant GROS_g03104 showed that the eggshell annexin has different conformations depending on presence or absence of calcium, confirming that at least one of these sites remains functional despite the disruption of another predicted site by the insertion (Fig 5). Later column elution times relate to smaller globular size, suggesting that in the absence of calcium the annexin is in a potentially relaxed state. Protein transition between these states was reversible. Notably, in the presence of host root diffusates there was a tendency for the annexin to maintain a larger globular state. Addition of 1 mM CaCl_2_ to the TRD did not cause the protein to shift to the calcium bound state. Column elution times suggested that ligand binding to the annexin did not change the protein multimeric state. This same trend was seen both on standard gel filtration and analytical columns. Both columns were unable to clearly differentiate changes in globular protein shape in the presence of host versus non-host root diffusates.

The calcium binding capacity of host root diffusates was tested in order to ensure that the diffusates themselves do not remove calcium from solution, explaining the change in annexin response to calcium in the presence of root diffusates. Patton-Reeder indicator (Calconcarboxylic acid) did not release calcium in the presence of TRD suggesting that root exudates do not have a strong ability to bind calcium directly. Change in annexin response to calcium in the presence of TRD therefore suggests that one or more components present in TRD directly interact with the *G*. *rostochiensis* eggshell annexin, inhibiting or competing with regular annexin-calcium binding.

While gel filtration is informative for large scale morphology changes, it gives little information other than abundance of the globular size of the protein. Near UV circular dichroism (CD) was used to interpret changes in recombinant annexin tertiary structure in response to presence or absence of a ligand [28]. These data confirm that the conformational change seen during gel filtration in response to the presence of calcium is not due to a drastic change in the annexin tertiary structure (Fig I in S1 Data). However, traces shift depending on the presence or absence of calcium, reflecting the gel filtration data. No changes in tertiary structure were recorded when in the presence of host vs non-host root diffusates.

### Annexin-lipid binding

Membrane lipid strips were used to identify changes in annexin-lipid interactions in response to the presence and absence of calcium and in the presence of host or non-host root diffusates. Calcium is present in all root diffusates so to quantify this, and allow normalisation in terms of calcium concentration, samples of TRD and GRD were analysed by inductively coupled plasma optical emission spectrometry (ICP-OES). These showed that the non-host exudate GRD had almost double the amount of calcium compared to TRD. Therefore, lipid blots were tested in 20% TRD or 10% GRD, normalising for calcium abundance following ICP-OES.

**Fig 6:**
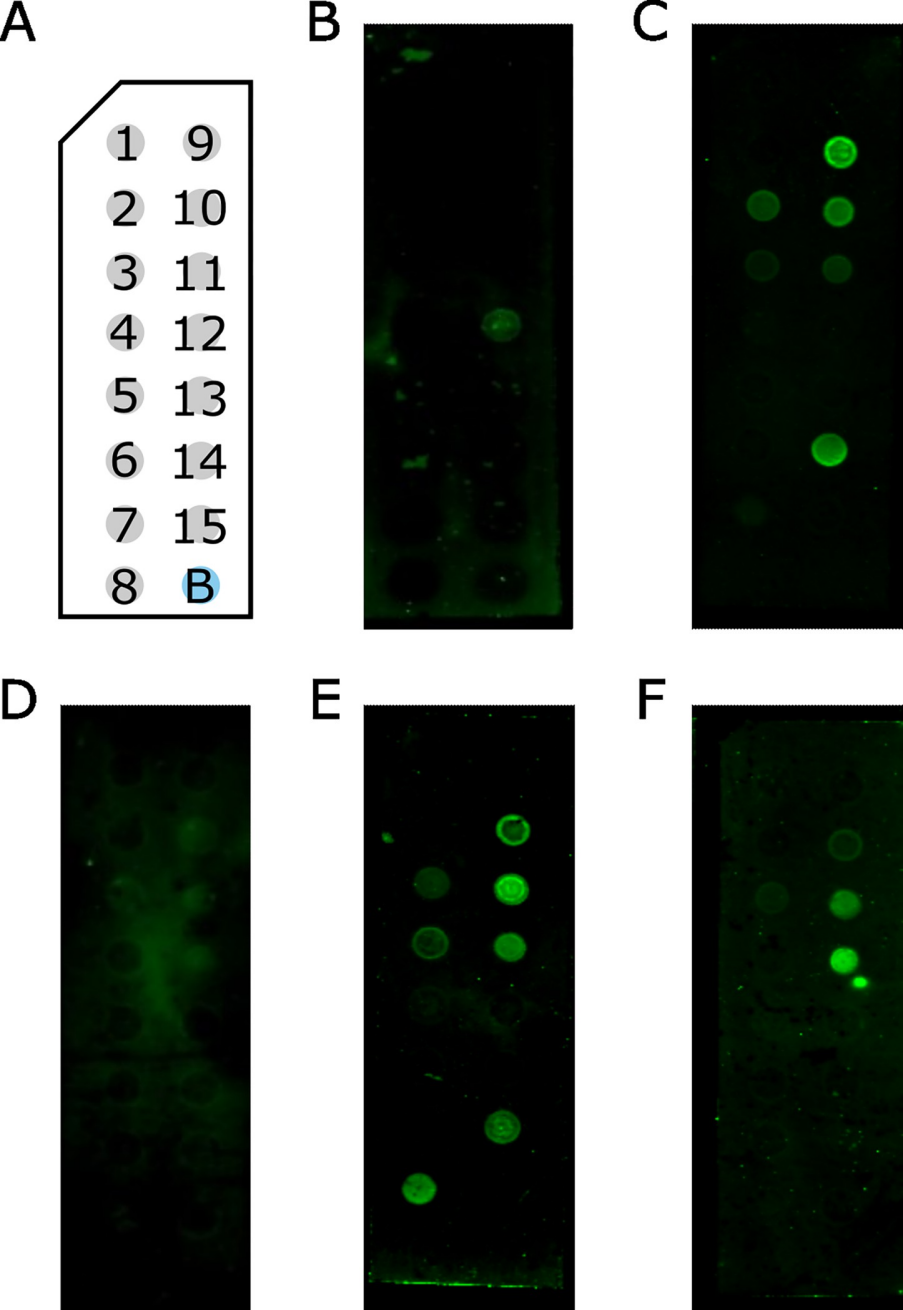
Annexin-lipid binding. (A) Membrane Lipid Strip (Echelon) schematic. 1 –Triglyceride, 2 –Diacylglycerol (DAG), 3 –Phosphatidic acid (PA), 4 –Phosphatidylserine (PS), 5 –Phosphatidylethanolamine (PE), 6 –Phosphatidylcholine (PC), 7 –Phosphatidylglycerol (PG), 8 –Cardiolipin, 9 –Phosphatidylinositol (PI), 10 –Phosphatidylinositol-4-phosphate (PIP), 11 –Phosphatidylinositol-4-5-bisphosphate (PIP_2_), 12 –Phosphatidylinositol-3-4-5-trisphosphate (PIP_3_), 13 –Cholesterol, 14 –Sphingomyelin, 15–3-sulfogalactosylceramide (sulfatide), B–Blank. (B) Annexin with 1 mM EDTA, low-level binding to PIP_3_. (C) Annexin with 1 mM CaCl_2_ binding to PIP, PIP_2_, PIP_3_, PA, PS & sulfatide. (D) Annexin with 20% TRD and 1 mM EDTA showing very low-level binding to PIP, PIP_2_, PIP_3_, PA. (E) Annexin with 20% TRD binding to PIP, PIP_2_, PIP_3_, PA, PS, sulfatide & cardiolipin. (F) Annexin with 10% GRD binding to PIP_2_, PIP_3_ with low-level binding to PIP & PA.

Recombinant eggshell annexin has a native affinity for negatively charged lipids, binding PIP_3_ in the absence of Ca^2+^ (Fig 6B). In the presence of calcium, the typical annexin preference for negatively charged lipids was amplified with clearer binding to almost all lipids carrying a negative charge on the blot (Fig 6C). As observed when EDTA alone was present, there is little visible binding to any lipids when the recombinant annexin is exposed to EDTA-treated TRD (Fig 6D). However, in the presence of untreated TRD, lipid binding patterns were changed, with binding to the same lipid species observed in the presence of 1 mM CaCl_2_ but with additional binding to cardiolipin (Fig 6E). This additional binding pattern was not replicated in the presence of non-host root exudates (Fig 6F). The presence of host root exudates therefore specifically changes the ability of the annexin to bind to lipids while no such changes are seen in the presence of non-host exudates.

## Discussion

Here we report the identification of the first eggshell protein of any parasitic nematode and show that this protein responds to the presence of host derived chemical cues. Knockout studies also show that this protein plays a role in the nematode’s ability to control the permeability of its eggshell.

Annexin sequences and structures are normally conserved, reflecting a conserved biochemical function. However, the annexin identified and localised to the *G*. *rostochiensis* eggshell contains a PCN specific motif that is predicted to disrupt one of the predicted calcium binding sites. Given that calcium binding is known to impact the structure and function of other annexins, the presence of a novel motif in this region is likely to be significant. Similarly localised insertions can not be seen in orthologues of this protein in other cyst nematodes species that hatch in response to the presence of a host (*H*. *schachtii* and *H*. *glycines*) [14]. Although, the cereal cyst nematode, *H avenae*, does have an insertion at this location, the length and sequence of the insertion is different in this protein. Hatching in *H*. *avenae* is associated with cold temperatures required to break dormancy. This could suggest that the motif insertion here regulates dormancy. Alternatively, motif insertions at this position could alter specific ligand binding affinity, reflecting requirements for change in cationic presence during hatching. Alternatively, they may allow binding of a ligand present in host exudates in a way that allows annexin structure to change in response to the presence of these ligands rather than calcium. The lack of direct calcium binding by TRD and changes in annexin-calcium binding in the presence of TRD suggests that a component of TRD directly interacts with the eggshell annexin. However, it is still unclear whether TRD binding occurs at a calcium binding site, the unique *Globodera spp*. motif or whether the calcium binding is altered through a different interaction.

Lipid binding assays showed the annexin has the typical preference of this type of protein for negatively charged lipids. Under consistent calcium concentrations there was a much higher lipid affinity, and a greater range of lipids bound, in the presence of host compared to non-host RD. This is critical as it shows that the presence of host signals changes a key biological property of the protein, linking this protein to the *G*. *rostochiensis* hatching cascade. It is worth noting that lipids bound on these blots may not reflect lipids present in the eggshells of *G*. *rostochiensis* and are instead an expression of the annexin’s preference for the presented lipids under certain condition. All of the characterisation presented here was undertaken using recombinant protein. Potato cyst nematodes are small and impossible to grow in the quantities that would be needed to repeat this work using purified protein. However, the annexin is not secreted and previous structural analyses have been performed using recombinant proteins. We are therefore confident that the data presented here reflect the properties of the protein in the nematode itself.

It is likely that the eggshell annexin is located at the lipid membranes that form the eggshell permeability barrier. This is consistent with the phenotype seen after RNAi, where a decrease in the eggshell annexin seemingly increased eggshell permeability. PCN cysts are dried as part of the normal harvesting process and this normally has no adverse effect on the ability of the nematode to survive. Again, this reflects the biological function of this life stage which is a survival stage that allows the nematode to persist in varied conditions until the presence of a new host plant is detected. When cysts are collected and stored in dry conditions, the increase in eggshell permeability due to the absence of annexin may enable water to evaporate away allowing partial desiccation of juveniles, reducing their overall size inside the eggshell and reducing their viability. Annexin positioning within the permeability barrier could be as suggested by Langen *et al*. [29] where under certain conditions annexin monomers become tightly packed and inserted across the phospholipid bilayer. When in the opposing interaction state, the annexin is removed from the bilayer, increasing membrane disorder and permeability. Tightly packed and relaxed conformational states such as this are mirrored in data collected here during size exclusion chromatography and circular dichroism. However, we were unable to detect significant changes to tertiary structure using these techniques, suggesting that changes induced by exposure to root exudates are more subtle and will require more detailed and sensitive analysis.

Our data are consistent with two potential models for the control of hatch. Following analysis of the biochemistry of hatching in *Ascaris suum* eggs, it was suggested that tori could be formed by ascarylose headgroups of ascaroside glycolipids [30]. It was suggested that these tori could form pores allowing trehalose to escape from within the eggshell permitting full hydration of the juvenile. Release of trehalose from the eggshell is also known to be part of the hatching process in PCN, suggesting that sizeable pores need to be formed to allow release of trehalose from the perivitelline fluid. Although further work is required to determine whether GROS_g03104 can natively form multimers as seen in other annexins (*e*.*g*., [31]) it is possible that a ligand-induced change in the interaction of GROS_g03104 could allow a multimeric pore to form, or open, that would allow trehalose to passively escape across the membrane. However, it is clear that this multimerisation is not spontaneous upon exposure to suitable host root diffusates given the data shown in Fig 5.

An alternative model, which does not rely on the formation of annexin pores large enough to allow passage of trehalose, can also be proposed. In this, PCN eggshell permeability barriers (lipids) are bound by annexins. Upon exposure to host root diffusates, these eggshell annexins change conformation and the range of lipids that they bind is modified. This change in lipid binding may increase lipid disorder causing the barrier to become permeable. This model may be consistent with the increased hatch seen when expression of the annexin encoding gene is reduced by RNAi. The *Globodera spp*. specific unique motif on one of the calcium-binding sites within the annexin is likely to play a critical role in the way in which this protein responds to host root diffusates, either directly or through changing the way in which the annexin binds to calcium in the presence of host root diffusates.

Although this work represents a significant advance, obtaining a crystal structure for this protein is required for full elucidation of the function of the annexin. Crystallisation in the presence and absence of host diffusates will confirm how the annexin responds to the host. Similarly, investigating ligand interaction will allow identification of alteration to binding capacity caused by the *Globodera spp*. unique motif. With widespread reduction in nematicides across UK and EU markets further work identifying functionality of the unique motif could facilitate creation of an incredibly well-targeted novel chemical control. Furthermore, it is hoped that similar studies on parasitic nematodes that do not exhibit hatching in response to specific host root diffusates will highlight other hatching mechanisms.

## Materials and methods

### Biological material

*Globodera rostochiensis* was maintained in a glasshouse on the susceptible potato (*Solanum tuberosum*) cultivar Desiree. Tomato root diffusate (TRD) was produced by washing soil from the roots of tomato plants (cv. Moneymaker) and leaving them in distilled H_2_O overnight at room temperature. The following day root diffusate was filtered through a 0.22 μm filter to remove particulates and potential sources of contamination. Filtered RD was stored at 4°C. Non-host (gooseberry) root diffusate (GRD) was collected and stored in the same way. Where root diffusates were to be used comparatively, samples of root diffusate were sent for inductively coupled plasma optical emission spectrometry (ICP-OES) analysis.

For purification of eggshells, around 50 *G*. *rostochiensis* cysts were placed in 1 mL of dH_2_O then transferred to a general-purpose Potter-Elvehjem homogeniser (GPE Scientific). The cysts were crushed and the resulting suspension was passed through two mesh sieves with gap widths of 50 μm and 38 μm to remove cyst fragments. An ultrasonic water bath (Grant ultrasonic bath MXB22) cooled to 4°C was used to break open the eggshells. Eggshells were separated from J2 and any remaining cyst debris by density centrifugation in sodium-potassium tartrate tetrahydrate. This method was partially modified from Clarke, Cox & Shepherd [32] by increasing the centrifugation time from 1 to 2 minutes and increasing the sonication time depending on the number of visualised burst eggshells.

### Protein extraction, identification and orthologue information

Purified eggshell samples were washed twice in sterile distilled water. Proteins were extracted from eggshells by shaking in 80:20 MeOH:H_2_O overnight. The methanol was evaporated and eggshells were resuspended in SDS-PAGE sample buffer, heated to 95°C and run into denaturing gels. The gel was stopped before proteins separated and stained with Coomassie blue. The stained band was excised from the gel and sent for mass spectrometry analysis (University of St. Andrews) (Fig B in S1 Data). Orthologous protein sequences from other nematode species for alignments were gathered from parasite.wormbase.org. Pairwise alignments were carried out through Jalview (2.11.2.5) using the MUSCLE web server at default settings (Fig C in S1 Data).

### Cloning and expression of recombinant protein

The gene encoding annexin GROS_g03104 was amplified from *G*. *rostochiensis* cDNA. Primers were designed to amplify the full coding region of the gene with the addition of appropriate restriction enzyme sites allowing cloning into the pEHISTEV vector [33] (Fig D in S1 Data). Plasmids containing the insert were transferred to *E*. *coli* (Rosetta 2) for expression and 10 mL starter cultures were grown overnight. Up to 5 L of LB was inoculated with starter cultures and after reaching an optimal density expression was induced with 1 mM IPTG and carried out at 18°C overnight. The resulting cell culture was pelleted, resuspended in lysis buffer (50 mM Tris pH 7.5, 0.3 M NaCl, 10 mM imidazole) and lysed by sonication. Lysed cell debris was separated from soluble proteins by centrifugation. Recombinant proteins, tagged with a 6xHis tag, were purified using a nickel column (GE Life Sciences). Following equilibration, the soluble fraction was run through an ÄKTA purification system. After column washing (lysis buffer, 50 mM imidazole), proteins bound to the column were eluted on a gradient (lysis buffer, 250 mM imidazole). Fractions from column purifications were monitored via SDS-PAGE. HIS tags were cleaved using HIS-tagged TEV produced in-house and run through a nickel column once more to purify cleaved protein. Buffer exchange using dialysis membrane was used to match the proteins’ buffer to the application downstream. If not used immediately, protein was kept in 50 mM HEPES (pH7.8), 0.1 M NaCl and 10% glycerol, snap frozen and stored at -80°C.

### Antibody synthesis and immunolocalisation

Unique immunogenic peptides within the target protein were identified by Eurogentec. The selected peptides were identified as being specific to GROS_g03104 using a BLAST search against the *G*. *rostochiensis* genome sequence. The peptide (N-^166^RDESWNTDPLRANMV^180^-C) was used to raise anti-peptides in rabbits using the 28-day ‘speedy’ protocol. The antipeptide was tested for reactivity against recombinant GROS_g03104 (Fig E in S1 Data).

For immuno-localisation, purified eggshell samples were fixed in 4% paraformaldehyde overnight. Fixative was removed by washing twice with Phosphate Buffered Saline (PBS). Eggshells were permeabilised by incubating in PBS + 0.1% Triton-X 100 followed by two PBS washes to remove Triton-X. Non-specific binding was blocked by incubating in 2% bovine serum albumen (BSA) for 1 hour at room temperature with gentle agitation. This was followed by 3 further washes in 0.1% BSA. Primary antibody solution (1:1000) in 0.1% BSA was applied to eggshells for 2 hours at room temperature. Primary antibodies were removed by washing samples in 0.1% BSA 6 times, changing the solution every 10 minutes. Antibodies were localised using Alexa Fluor Plus 488 anti-rabbit antibodies. Secondary antibodies were incubated with eggshells for 1 hour at room temperature. Six further 10-minute washes in PBS were used to remove any unbound secondary antibody. Rabbit pre-immune serum was used as a negative control.

Samples were imaged using a Zeiss LSM710 laser scanning microscope mounted on an AxioImager z2 motorised upright microscope. To overcome issues with autofluorescence, eggshells that had not been exposed to any antibodies were first observed under the microscope. Computational gain in channel 488 was subsequently reduced to a level where eggshell auto fluorescence was only just visible effectively zeroing the microscope to allow any additional fluorescence, resulting from secondary antibodies, to be seen. The same microscope settings were used for imaging all eggshells. Images were simultaneously captured in channel 516 to confirm that computational gain had only been reduced in one channel. Although images have been brightened for publication, all images were treated simultaneously and uniformly.

### RNAi

Hairpin RNA was created by cloning a short, gene-specific sequence into vectors pHannibal and later, pART-27. pART-27 containing the GROS_g03104 forward and reverse sequences flanking the PDK intron was transformed into electro-competent *Agrobacterium tumefaciens* strain AGL1 and grown in liquid culture at 28°C overnight.

Internodes were cut from 4-6-week-old Desiree plantlets, covered with transformed *Agrobacterium* and gently rocked at room temperature to ensure even coverage of bacteria onto plant tissue. Internodes were transferred onto callus induction media plates (1 L MS30 media with 2.5 mg Zeatin Riboside, 0.2 mg naphthalene acetic acid and 0.02 mg gibberellic acid). After 2–3 days internodes were transferred to agar plates containing timentin and kanamycin for 2 weeks. Surviving calli were transferred to shoot promotion media plates (hb2) and after 2 weeks shoots were excised and transferred to MS30 root induction media plates containing the same antibiotics. Once roots had begun to form, leaf material was removed to check for the presence of the GROS_g03104 hairpin. gDNA was extracted using the DNeasy Plant Mini Kit (Qiagen) using manufacturers guidelines. PCR using gDNA and GROS_g03104 FR-RF primers (Fig D in S1 Data) was used to identify successfully transformed plants. Stem tips were cut from RNAi hairpin containing candidates and planted into insecticide free compost.

Semi quantitative RT-PCR was used to compare expression of the RNAi construct in each plant line. Four plant lines were taken that showed a range of expression levels of the shRNA, along with one control line. 20 cuttings were taken from each mother plant. The cut end was dipped in rooting powder and placed into a peat pellet (Jiffy). Cuttings were left well-watered for 2 weeks until roots appeared from the base of the pod.

Inoculations were carried out using 20 handpicked cysts of *G*. *rostochiensis* of uniform shape and size per plant. Rootrainers (Haxnicks) were filled with 50:50 autoclaved sand:loam and watered. A hole was formed in the sand:loam using a 5 mL pipette tip dipped up to the 3 mL mark. 20 cysts were dropped into the hole using a clean 5 mL pipette tip as a funnel. The hole was filled in and a peat pod (Jiffy) containing the growing cutting was placed on top of the sand:loam allowing roots to grow through the root trainer.

### qPCR and cyst extraction

Levels of GROS_g03104 mRNA in *G*. *rostochiensis* females grown on the transgenic lines were quantified using qPCR. Seven weeks after inoculation, 5 females were removed from the roots of 3 daughter plants per plant line and frozen in liquid nitrogen. Nematode RNA was extracted using an RNeasy Plant Micro kit (Qiagen) using the manufacturer’s protocol. cDNA was synthesised using 80 ng of RNA for each sample. A standard PCR was carried out to confirm that there was no amplification from RNA using the 3104RNAi_F/R primer pair. Similarly, a PCR was carried out on newly synthesised cDNA to check that the primer pairs specifically amplified a product of the anticipated size. qPCR reactions consisted of 2 μL template cDNA, 6 μL UV treated SDW, 1 μL of both 3104RNAi_F and 3104RNAi_R primers (10 μm) (Fig D in S1 Data) and 10 μL 2x LightCycler FastStart DNA master mix SYBR green I (Roche). For each sample an additional reaction was carried out using primers for the housekeeping Glyceraldehyde 3-phosphate dehydrogenase (GAPDH) gene to confirm that cDNA quantity did not significantly vary between samples. After an initial denaturing step at 95°C for 10 minutes, cycling conditions repeated 40 cycles of denaturing at 95°C for 10 seconds, annealing at 54°C for 10 seconds and elongating at 72°C for 30 seconds. qPCR was carried out using an LC480 LightCycler (Roche) in LightCycler 480 multiwell 96 white plates. qPCR data were normalised against the housekeeping GAPDH gene and fold change was represented as ΔCT.

Cysts were extracted from the soil using standard protocols after plants had been allowed to die back and dry. Cysts were stored at room temperature.

### Hatching assays

Hatching assays were completed in 24-well plates. 15 cysts from each line were placed in water overnight. Cysts were separated into 5 replicates of 3 cysts per well. Water was replaced with root diffusate and plates were covered, sealed and stored at 18°C. Hatched nematodes were counted weekly for 4 weeks.

### Size exclusion chromatography

A Superose 6 Increase 10/30 gel filtration column (GE Healthcare) was used for size exclusion chromatography. Columns were loaded and washed using an ÄKTA purification system. Approximately 5 mg of protein was used for testing protein changes in the presence of CaCl_2_ and EDTA while 2.5 mg of protein was used when the column was equilibrated with TRD. Columns were equilibrated with 150 mM NaCl containing either 1 mM CaCl_2_, 1 mM of both EDTA and EGTA or 20% TRD or a combination of these for the appropriate conditions. Column loads and elution were carried out under constant pump speeds.

### Lipid dot blots

Membrane lipid strips were purchased from Echelon Biosciences. Membranes were blocked in 1% fatty acid free BSA in PBS for 1 hour followed by one 5-minute wash in PBS. Membranes were then transferred to TTBS (50 mM Tris pH7.5, 150 mM NaCl, 0.1% Tween 20) containing either 1 mM EDTA, 1 mM Calcium, 20% RD or 1 mM EDTA & 20% RD and recombinant GROS_g03104 at a final concentration of 6 μg/mL. Blots were left agitating overnight at 4°C. Membranes were washed with PBS-Tween20 (0.1%). Washes were repeated 3 times at room temperature for 5, 10 then 15 minutes. Blots were transferred to PBS containing 0.5% BSA with 1:5000 primary antibody (as used above) and left agitating at room temperature for 2 hours. 3 more PBS-T washes out of the primary antibody were followed by addition of the secondary goat anti-rabbit 800 (IRDye, Li-Cor) in the same buffer for 1.5 hours at room temperature. Membranes were washed 3 times in PBS-T and imaged using a Li-Cor Odyssey.

## Supporting information

S1 Data Fig A. Eggshell purification. Fig B. Mass spectrometry data. Fig C. Annexin interspecies alignment. Fig D. Primers. Fig E. Antibody information. Fig F. qPCR data. Fig G. RNAi eggshell measurements. Fig H. Pre-diapause hatching data. Fig I. Circular dichroism. Figure legends for S1 Data. Fig A in S1 Data. A–Eggshell/nematode sample after cysts are crushed with a tissue homogeniser. B–Eggshell lysis after a period in the ultrasonic water bath. C–purified eggshells after sodium-potassium tartrate gradient density centrifugation. Images taken on a moticam microscope camera attached by a 0.5x magnification mount seated above a 40x objective lens. Fig B in S1 Data. Mass spectrometry data of proteins extracted from eggshells. Fig C in S1 data. Alignment of GROS_g03104 against orthologues in other plant parasitic nematodes. Free-living nematodes from the genus *Caenorhaditis* were included as non-parasitic nematode comparatives. *D*. *melanogastor* provides a non-nematode outlier. GROS_g03104 orthologues were identified through parasite.wormbase. Sequence alignment was completed using the MUSCLE server with default settings. CLUSTAL X colours highlighting amino acid profile were added using Jalview (2.11.2.5). Fig D in S1 Data. Primers used in this study. Fig E in S1 Data. Information on region of protein used for antibody production. Fig F in S1 Data. qPCR data from developing *G*.*rostochiensis* females reflecting change in expression of GROS_g03104 following host induced gene silencing (HIGS). EV—empty vector, 2,6,7,20 represent plant lines determined to be expressing the RNAi construct. Variation in success of HIGS can be seen between females multiplying on the same plants lines. Fig G in S1 Data. Cyst diameter measurements from *G*. *rostochiensis* populations grown on RNAi plant lines (6, 20) or control plant lines not expressing the HIGS construct. Population A2012 represents the initial population used to infect RNAi plant lines. Diameters were measured using imageJ measuring from left to right lateral sides of the cyst. ANOVA including 2nd generation EV and HIGS shows no significant variance between the diameter of these populations. Fig H in S1 Data. Hatching of juveniles from RNAi treated and control eggs. Fig I in S1 Data. Circular dichroism using recombinant GROS_g03104 under various conditions. Recombinant annexin was diluted in 50 mM Tris (pH7.5), 100 mM NaCl, 10% glycerol and either 1 mM CaCl_2_, 1 mM EDTA, tomato or potato root diffusates. 2 mL sample sizes including 1 mg/mL protein were used for each sample. Samples were loaded into a 10 mm cuvette and readings were taken between 260 nm-320 nm using a Mos-500 circular dichroism spectrophotometer (Biologic) coupled with and ALX-300 lamp unit running above 145 W. Slit size was set to 2 nm and readings were taken stepwise every 0.25 nm. No large variation in CD traces can be seen for the recombinant annexin under any conditions suggesting that neither host RD or the presence/absence of calcium drastically changes the annexin tertiary structure.
(XLSX)
